# Safety and efficacy of fecal microbial transplantation for the prevention and treatment of acute graft-versus-host disease: a meta-analysis

**DOI:** 10.3389/fmicb.2026.1802260

**Published:** 2026-05-22

**Authors:** Lijuan Zhang, Shuxiang Lin, Bin Zu, Zhengrong Chen, Shufa Li, Wei Lin, Tian Dong, Zehui Chen

**Affiliations:** 1 Department of Hematology, West China Hospital, West China Xiamen Hospital, Sichuan University, Xiamen, China; 2 Department of Pathology, Affiliated Hospital of Putian University, Putian, China; 3 Department of Gastrointestinal Surgery, Affiliated Hospital of Putian University, Putian, China; 4 Department of Anorectal Surgery, Affiliated Hospital of Putian University, Putian, China; 5 Department of Hematology, West China Hospital, Sichuan University, Chengdu, China

**Keywords:** aGVHD, allo-HSCT, FMT, prevention, treatment

## Abstract

**Objective:**

In recent years, fecal microbiota transplantation (FMT) has been increasingly investigated for the prevention and treatment of acute graft-versus-host disease (aGVHD). Nevertheless, its clinical efficacy remains uncertain. Therefore, this study aims to systematically evaluate the clinical efficacy of FMT in preventing and treating aGVHD.

**Methods:**

We systematically searched Cochrane Library, PubMed, Embase, and Web of Science from inception to October 2025 for studies comparing FMT with conventional regimens (corticosteroids and/or immunosuppressants) for aGVHD prevention and treatment. All statistical analyses were performed using RevMan 5.4.1 and Stata 16.

**Results:**

Six studies involving 262 patients were included. Among them, 85 patients received FMT for aGVHD prevention, 65 received conventional prophylaxis, 68 received FMT for Gastrointestinal aGVHD (GI-aGVHD) treatment, and 44 received conventional treatment for GI-aGVHD. Meta-analysis showed no significant difference in the incidence of aGVHD between the FMT and conventional groups [odds ratio (OR) = 1.30, 95% confidence interval (CI) = 0.10-16.72, *p* = 0.84]. However, the FMT group demonstrated significantly higher 14-day and 30-day complete response (CR) rates, as well as 14-day clinical response rates, in patients with GI-aGVHD compared to the conventional group (OR = 8.54, 95% CI = 2.49–29.29, *p* = 0.0007; OR = 8.44, 95% CI = 2.98–23.96, *p* < 0.0001; OR = 4.66, 95% CI = 1.73–12.55, *p* = 0.002). No significant differences were observed in the incidence of bacteremia or sepsis between the two groups (OR = 0.37, 95% CI = 0.13–1.01, *p* = 0.05; OR = 0.38, 95% CI = 0.11–1.33, *p* = 0.13). Additionally, the abundances of Bacteroides and Bifidobacterium were significantly higher in the FMT group than in the conventional group [standardized mean difference (SMD) = 1.59, 95% CI = 0.15–3.03, *p* = 0.03; SMD = 1.01, 95% CI = 0.41–1.60, *p* = 0.0009].

**Conclusion:**

FMT showed favorable effects in improving clinical symptoms of GI-aGVHD and increasing the abundance of beneficial gut bacteria, and no increased risk of bloodstream infection was observed. These findings suggest that, for patients with established GI-aGVHD who may respond poorly to conventional regimens, FMT can serve as an effective adjunctive or salvage treatment. However, no significant advantage was observed for FMT in preventing aGVHD.

## 1 Introduction

Acute graft-versus-host disease (aGVHD) represents a major complication and cause of mortality following allogeneic hematopoietic stem cell transplantation (allo-HSCT; Takamatsu et al., 2022). Gastrointestinal aGVHD (GI-aGVHD) is particularly common and severe, characterized primarily by symptoms such as abdominal pain, diarrhea, and hematochezia (Chirletti et al., 1998). Currently, first-line treatment for aGVHD mainly consists of corticosteroids, but drug resistance frequently develops (Hematopoietic Stem Cell Application Group, Chinese Society of Hematology, and Chinese Medical Association, 2014). For steroid-refractory aGVHD, second-line treatment largely relies on novel immunomodulatory agents, such as ruxolitinib (a JAK inhibitor) and anti-CD25 monoclonal antibodies, which exert anti-GVHD effects by targeting inflammatory signaling pathways. Nevertheless, the clinical efficacy of these agents varies considerably, and the risk of severe infectious complications (e.g., invasive aspergillosis) has been widely reported. Some patients remain refractory or intolerant to these drugs, and a standardized second-line treatment regimen has not yet been established (von et al., 2018). Fecal microbiota transplantation (FMT), as an intervention that modulates the gut microbiome, offers an alternative second-line salvage option for these patients.

In recent years, studies have shown a close association between gut microbiota and aGVHD. During the pre-conditioning phase prior to allo-HSCT, radiochemotherapy and prophylactic antibiotic usage often lead to the disruption of intestinal microbiota diversity, with decreased abundance of beneficial bacteria such as Bacteroides, Lactobacillus, and Bifidobacterium, while opportunistic pathogens including Enterococcus, Akkermansia, and Clostridioides difficile relatively proliferate. This dysbiosis damages the intestinal mucosal barrier and activates inflammatory responses, thereby promoting the onset and progression of aGVHD (Kumari et al., 2019). Consequently, protecting and restoring a balanced gut microbiota holds promise as a novel strategy for preventing and treating aGVHD. FMT, which offers the advantages of rapidly restoring microbial diversity, re-establishing immune homeostasis, and repairing the intestinal barrier, has shown significant efficacy in patients with steroid-refractory GVHD in preliminary studies (Habibi and Rashidi, 2023).

Therefore, this study aims to systematically review published research on the use of FMT for the prevention and treatment of aGVHD. We will employ meta-analysis to evaluate its efficacy—including the incidence of aGVHD, complete response (CR) and clinical response [CR + partial response (PR)] in GI-aGVHD, and changes in gut microbiota abundance—as well as its safety, particularly regarding the risk of infections. The findings are expected to provide higher-level evidence to inform clinical decision-making.

## 2 Materials and methods

### 2.1 Search strategy

This study strictly adhered to the Preferred Reporting Items for Systematic Reviews and Meta-Analyses (PRISMA) guidelines. Two independent researchers systematically searched the PubMed, Embase, Cochrane Library, and Web of Science databases. The search period spanned from the inception of each database to October 2025, with search terms limited to “Title/Abstract.” The main search strategy was as follows: (((((((((fecal[Title/Abstract])) OR(fecal[Title/Abstract])) OR (feces[Title/Abstract])) OR (feces[Title/Abstract]))OR (gut[Title/Abstract])) OR (intestin^*^[Title/Abstract]))) OR(((microbi^*^[Title/Abstract]) OR (bacteri^*^[Title/Abstract]))) OR(((((transplant^*^[Title/Abstract]) OR (transfer[Title/Abstract])) OR(therapy[Title/Abstract])) OR (biotherap^*^[Title/Abstract])))) OR(FMT[Title/Abstract]) OR (fecal microbiota transplantation[MeSH Terms])AND(((((graft vs. host[Title/Abstract]) OR (graft vs. host[Title/Abstract])) OR(graft-versus-host[Title/Abstract])) OR (graft vs. host[Title/Abstract])) OR((disease[Title/Abstract]) OR (reaction[Title/Abstract]))) OR(GVHD[Title/Abstract]) OR (graft vs. host disease[MeSH Terms]). The search was limited to human studies without language restrictions. Additionally, the reference lists of included studies were manually searched to ensure comprehensive coverage.

### 2.2 Study inclusion criteria

Studies were included if they met the following criteria: (i) Participants: the study population consisted of either adults (aged ≥18 years) alone, or a mixed population comprising both pediatric (< 18 years) and adult patients. Participants were individuals in the peri allo-HSCT period, or patients with a confirmed and graded diagnosis of aGVHD according to the Mount Sinai Acute GVHD International Consortium (MAGIC) criteria (Schoemans et al., 2018). The diagnosis and severity grading of GI-aGVHD were defined according to the MAGIC criteria. (ii) Intervention: Prophylactic studies: FMT administered to peri allo-HSCT patients to prevent aGVHD. Therapeutic studies: FMT administered to patients with established aGVHD. (iii) Comparator: Prophylactic studies: Conventional prophylaxis regimens, blank control, or placebo control. Therapeutic studies: Conventional treatment regimens (corticosteroids and/or immunosuppressants), blank control, or placebo control. (iv) Outcomes: Studies from which data could be extracted or calculated, including the incidence of aGVHD, CR and PR rates at 14 and 30 days in patients with GI-aGVHD, incidence of bacteremia and sepsis, and gut microbiota abundance. CR was defined as a reduction of ≥500 mL in the mean daily stool volume within 3 days, or resolution of symptoms such as diarrhea, abdominal pain, and hematochezia (Qi et al., 2018; Zhao et al., 2021; Zheng et al., 2023). PR was defined as a reduction of < 500 mL in stool volume within 3 days, accompanied by alleviation of diarrhea, abdominal pain, or hematochezia. (v) Study Design: Randomized controlled trials (RCTs) and cohort studies.

### 2.3 Exclusion criteria

The exclusion criteria were as follows: (1) studies published in the form of reviews, case reports, or meta-analyses; (2) studies with unavailable or incomplete data that did not provide relevant outcomes; (3) non-human studies; (4) single-arm studies without control groups; (5) duplicate publications.

### 2.4 Data extraction

The following data were extracted: first author, year, country, number of enrolled patients, age, number of patients in the FMT group vs. the conventional regimen group, type of primary disease, FMT group protocol, control group protocol, incidence of aGVHD, CR and PR rates for GI-aGVHD at 14 and 30 days, incidence of bacteremia and sepsis, and metrics of gut microbiota abundance. Two independent researchers extracted raw data from box plots depicting gut microbiota abundance using WebPlotDigitizer and performed data transformation. All data were reviewed by a third researcher. Any discrepancies were resolved through discussion to reach a consensus (Schoemans et al., 2018).

### 2.5 Quality assessment

The quality of randomized controlled trials (RCTs) was assessed using the modified Jadad scale, with a total score of 7. Studies scoring ≥4 were considered high-quality (Jadad et al., 1996). The quality of the included cohort studies was evaluated using the Newcastle-Ottawa Scale (NOS), with a total score of 9. Studies scoring >6 were deemed high-quality (Stang, 2010). Two independent researchers reviewed and assessed the quality of the included studies. Disagreements were resolved through discussion or by a third researcher. The investigators were selected based on their relevant clinical expertise, prior FMT research experience, and absence of conflicts of interest.

### 2.6 Statistical analysis

Statistical analyses were performed using Review Manager software (version 5.4.1). For dichotomous variables, including the incidence of aGVHD, the CR and clinical response rate (CR+PR) of GI-aGVHD at days 14 and 30, and the incidence of bacteremia and sepsis, odds ratios (OR) with their 95% confidence intervals (CI) were calculated as the effect measure. For continuous variables, such as the abundance of Bifidobacterium and Bacteroides, the standardized mean difference (SMD) along with its 95% CI was used as the effect measure. Heterogeneity was assessed using the *I*^2^ statistic and Cochran's *Q* test. A random-effects model was applied if *I*^2^ > 50% or the *P*-value from Cochran's *Q* test was < 0.10, otherwise, a fixed-effects model was used (Bowden et al., 2011). The included studies consisted of those enrolling only adult patients (aged ≥18 years) and those enrolling a mixed population of pediatric (< 18 years) and adult patients. For studies that included both age groups, aggregate data were used for analysis when age stratified data were not available. To further explore the impact of age on the results, subgroup analyses were planned based on study population type (adult only vs. mixed population).

### 2.7 Sensitivity analysis

A sensitivity analysis was performed by sequentially excluding each included study and recalculating the pooled effect size based on the remaining studies. This approach was used to determine whether the magnitude and direction of the effect were significantly altered, thereby assessing the robustness of the overall results.

### 2.8 Publication bias

Publication bias was assessed using funnel plots. A symmetric funnel shape with evenly distributed scatter points suggested no obvious publication bias, whereas asymmetry indicated potential publication bias (Soeken and Sripusanapan, 2003). Furthermore, Egger's test was conducted using Stata 16. A *P*-value < 0.05 was considered indicative of significant publication bias (Irwig et al., 1997).

### 2.9 Quality of evidence

The quality of evidence for each outcome measure was comprehensively evaluated using the GRADEpro GDT software. This assessment included the evaluation of risk of bias, inconsistency in study results, indirectness of evidence, precision of effect estimates, and potential publication bias. The quality of evidence was categorized into four levels: high, moderate, low, and very low (Guyatt et al., 2008).

## 3 Results

### 3.1 Literature search results

**Figure 1:**
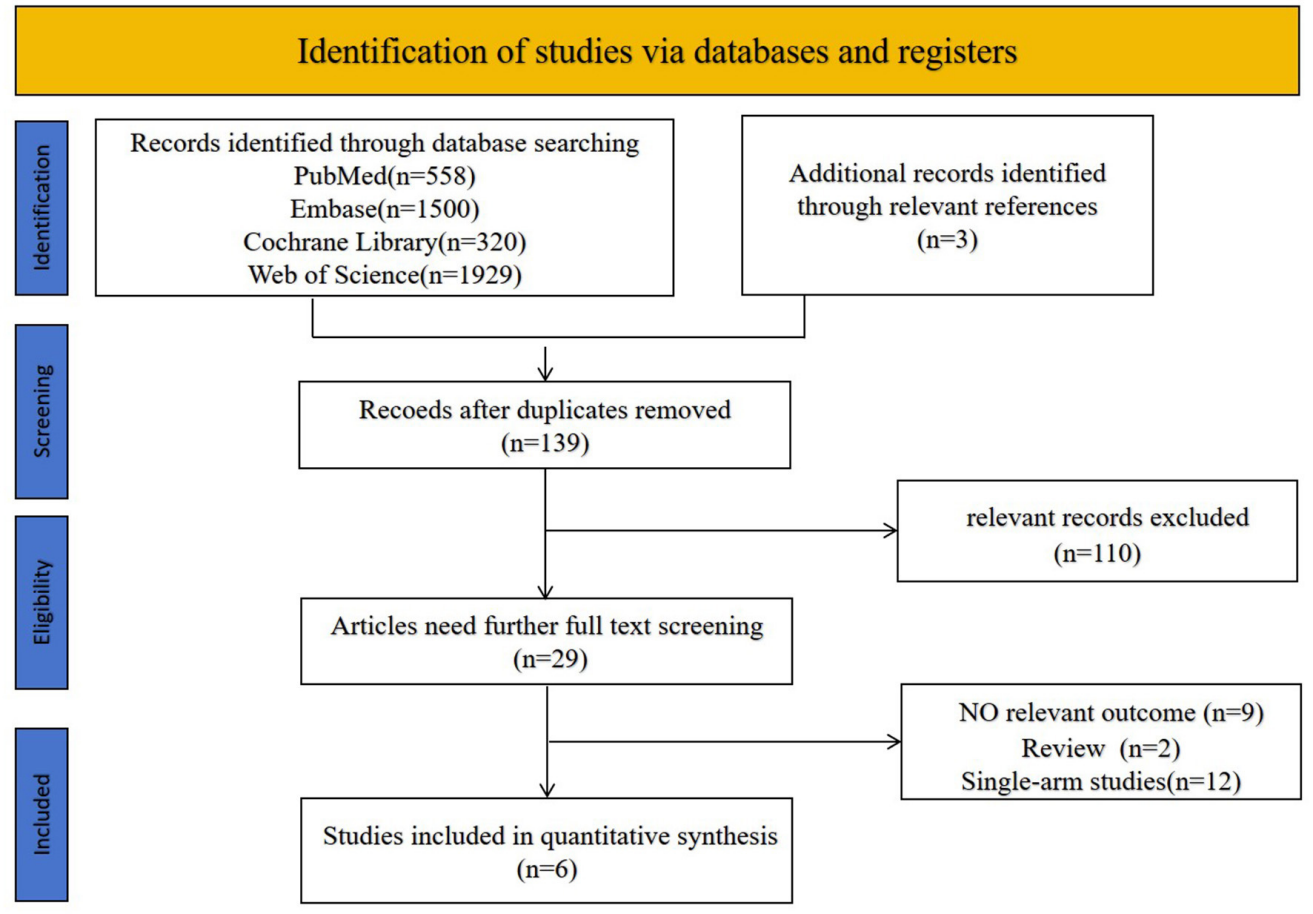
PRISMA flowchart.

The initial search strategy identified 4,307 studies. An additional 3 studies were identified by screening reference lists of relevant articles. After removing duplicates, 139 studies remained. Of these, 110 were excluded based on the screening of titles and abstracts. The remaining 29 studies underwent full-text review, resulting in the exclusion of 23 articles. Ultimately, 6 studies were included in the analysis (Figure 1).

### 3.2 Characteristics and quality

The six included studies comprised a total of 262 patients. This included 85 patients receiving FMT for aGVHD prevention and 65 patients receiving conventional regimens for aGVHD prevention, as well as 68 patients receiving FMT for GI-aGVHD treatment and 44 patients receiving conventional regimens for GI-aGVHD treatment. The included studies consisted of 3 RCTs and 3 cohort studies, all published between 2018 and 2024. Four studies were conducted in China, one in the United States, and one in Russia. The quality of the RCTs was assessed using the modified Jadad scale, while the cohort studies were evaluated using the NOS. The assessment results indicated that the overall quality of the included studies was moderate to high, suggesting a satisfactory level of reliability (Tables 1–3).

**Table 1:** Basic characteristics of included studies.

References	Country	Sample size FMT/control	Age FMT/control	Gender (male/female) FMT/control	Style of disease FMT/control	FMT	Control	Design
Rashidi et al. (2023)	USA	49/25	66.5 ± 10.6/67.9 ± 11.4	26/23 16/9	ALL: 33/20 MDS: 7/2 CLL/NHL: 3/1 Others: 6/2	Oral allogeneic FMT capsules	Placebo	RCT
Liu et al. (2024)	China	36/40	34(18–54)/32(18–64)	NR	NR	Oral autologous FMT capsules	Blank	RCT
Qi et al. (2018)	China	7/8	NR	3/5 6/2	CML: 1/1 AML: 2/2 MDS: 2/1 HCL: 1/1 ALL/LBL: 2/1 AA: 0/2	Nasoduodenal tune infuse allogeneic FMT	CG+IS	Cohort study
Zheng et al. (2023)	China	19/10	30(13–60)/41(17–61)	12/7 4/6	AML: 10/2 ALL: 2/3 CML: 1/0 MDS: 3/5 AA: 3/0	Oral allogeneic FMT capsules and nasoduodenal tube infuse allogeneic FMT	CG+IS	Cohort study
Zhao et al. (2021)	China	23/18	30(13–55)/31.5(13–59)	16/7 7/11	AML: 8/9 ALL: 3/2 MDS: 5/4 AA: 4/1 CML: 1/6 Others: 2/2	Nasojejunal or gastric tube infuse allogeneic FMT	IS	RCT
Goloshchapov et al. (2020)	Russia	19/8	NR	10/9 3/5	CML: 1/1 ALL: 5/4 AML: 5/1 MDS: 3/1 NHL: 1/1	Oral allogeneic FMT capsules and nasoduodenal tube infuse allogeneic FMT	CG+IS	Cohort study

ALL, acute lymphoblastic leukemia; MDS, myelodysplastic syndromes; CLL, chronic lymphocytic leukemia; NHL, non-hodgkin lymphoma; CML, chronic myeloid leukemia; AML, acute myeloid leukemia; HCL, hairy cell leukemia; LBL, lymphoblastic lymphoma; AA, aplastic anemia; FMT, fecal microbiota transplantation; NR, not reported; RCT, randomized controlled trial; CG, conventional glucocorticoids; IS, immunosuppressants.

**Table 2:** The modified Jadad scale of randomized controlled trials.

References	Random sequence production (2)	Allocation concealment (2)	Blind method (2)	Withdrawal (1)	Total
Rashidi et al. (2023)	2	2	1	1	6
Liu et al. (2024)	1	1	1	1	4
Zhao et al. (2021)	2	1	1	1	5

**Table 3:** The Newcastle-Ottawa Scale (NOS) of cohort studies.

References	Representativeness of the exposed cohort (1)	Selection of the non-exposed cohort (1)	Ascertainment of exposure (1)	Demonstration that outcome of interest was not present at start of study (1)	Compare ability of cohorts on the basis of the design or analysis (2)	Assessment of outcome (1)	Was follow up long enough for outcomes to occur (1)	Adequacy of follow up of cohorts (1)	Total
Qi et al. (2018)	0	1	1	1	1	1	1	1	7
Zheng et al. (2023)	1	1	1	1	2	1	1	1	9
Goloshchapov et al. (2020)	1	1	1	1	1	1	1	1	8

### 3.3 Meta-analysis results

#### 3.3.1 Prophylactic outcome

##### 3.3.1.1 Incidence of aGVHD

**Figure 2:**
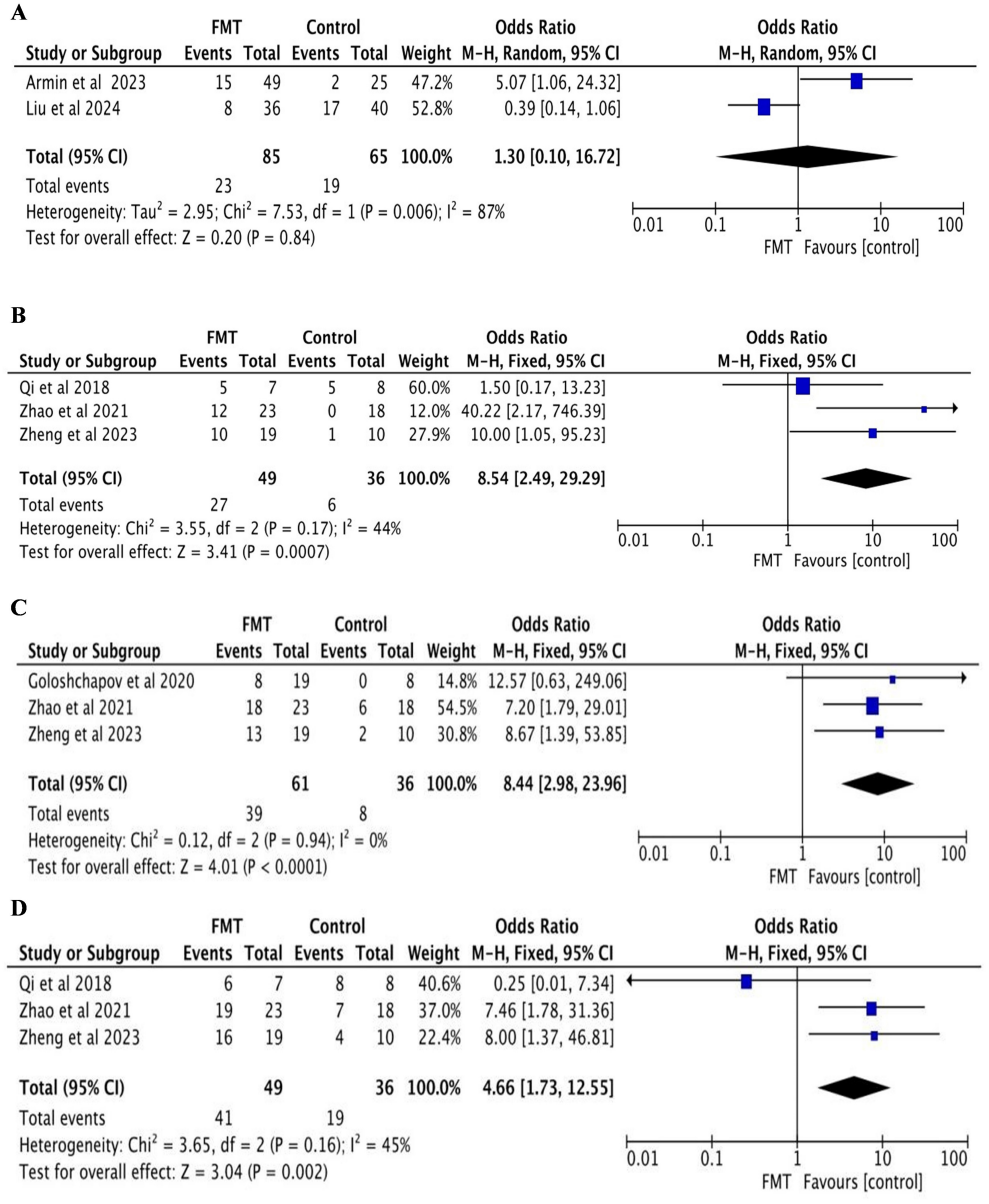
Clinical efficacy analysis: **(A)** incidence of aGVHD; **(B)** 14-day complete response; **(C)** 30-day complete response; **(D)** 14-day clinical response.

Two studies were included, both reporting the incidence of aGVHD (Rashidi et al., 2023; Liu et al., 2024). The FMT group comprised 85 patients, while the control group included 65 patients. Significant heterogeneity was observed among the studies (*p* = 0.006, *I*^2^ = 87%), and thus a random-effects model was applied. The meta-analysis demonstrated no significant difference in the incidence of aGVHD between the two groups (OR = 1.30, 95% CI = 0.10–16.72, *P* = 0.84; Figure 2A).

#### 3.3.2 Therapeutic outcomes

##### 3.3.2.1 14-day CR of GI-aGVHD

Three studies were included, all of which reported the CR of GI-aGVHD at 14 days (Qi et al., 2018; Zhao et al., 2021; Zheng et al., 2023). The FMT group consisted of 49 patients, and the control group included 36 patients. No significant heterogeneity was detected (*p* = 0.17, *I*^2^ = 44%), and a fixed-effects model was employed. The meta-analysis revealed that the CR rate in the FMT group was significantly higher than that in the control group (OR = 8.54, 95% CI = 2.49–29.29, *p* = 0.0007; Figure 2B).

##### 3.3.2.2 30-day CR of GI-aGVHD

Three studies were included, all reporting the CR of GI-aGVHD at 30 days (Zhao et al., 2021; Zheng et al., 2023; Goloshchapov et al., 2020). The FMT group included 61 patients, and the control group comprised 36 patients. No significant heterogeneity was observed (*p* = 0.94, *I*^2^ = 0%), and a fixed-effects model was utilized. The meta-analysis indicated that the CR rate in the FMT group was significantly higher than that in the control group (OR = 8.44, 95% CI = 2.98–23.96, *p* < 0.0001; Figure 2C).

##### 3.3.2.3 14-day clinical response of GI-aGVHD

Three studies were included, all of which reported the clinical response rate of GI-aGVHD at 14 days (Qi et al., 2018; Zhao et al., 2021; Zheng et al., 2023). The FMT group involved 49 patients, and the control group included 36 patients. No significant heterogeneity was found (*p* = 0.16, *I*^2^ = 45%), and a fixed-effects model was applied. The meta-analysis showed that the clinical response rate in the FMT group was significantly higher than that in the control group (OR = 4.66, 95% CI = 1.73–12.55, *p* = 0.002; Figure 2D).

##### 3.3.2.4 Bacteremia

**Figure 3:**
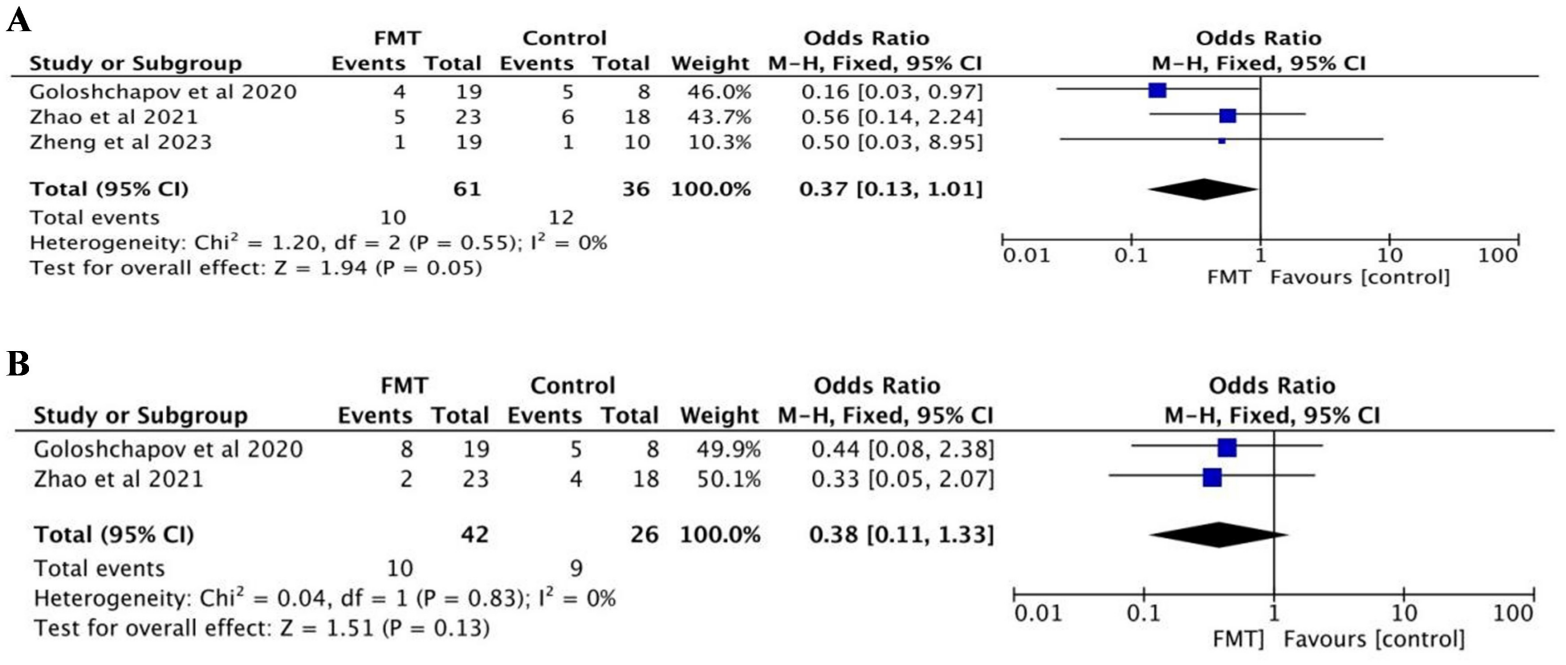
Safety analysis: **(A)** incidence of bacteremia; **(B)** incidence of sepsis.

Three studies were included, all reporting the incidence of bacteremia (Zhao et al., 2021; Zheng et al., 2023; Goloshchapov et al., 2020). The FMT group consisted of 61 patients, and the control group included 36 patients. No significant heterogeneity was detected (*p* = 0.55, *I*^2^ = 0%), and a fixed-effects model was used. The meta-analysis demonstrated no significant difference in the incidence of bacteremia between the two groups (OR = 0.37, 95% CI = 0.13–1.01, *p* = 0.05; Figure 3A).

##### 3.3.2.5 Sepsis

Two studies were included, both reporting the incidence of sepsis (Zhao et al., 2021; Goloshchapov et al., 2020). The FMT group comprised 42 patients, and the control group included 26 patients. No significant heterogeneity was observed (*p* = 0.83, *I*^2^ = 0%), and a fixed-effects model was employed. The meta-analysis revealed no significant difference in the incidence of sepsis between the two groups (OR = 0.38, 95% CI = 0.11–1.33, *p* = 0.13; Figure 3B).

##### 3.3.2.6 Abundance of bacteroides

**Figure 4:**
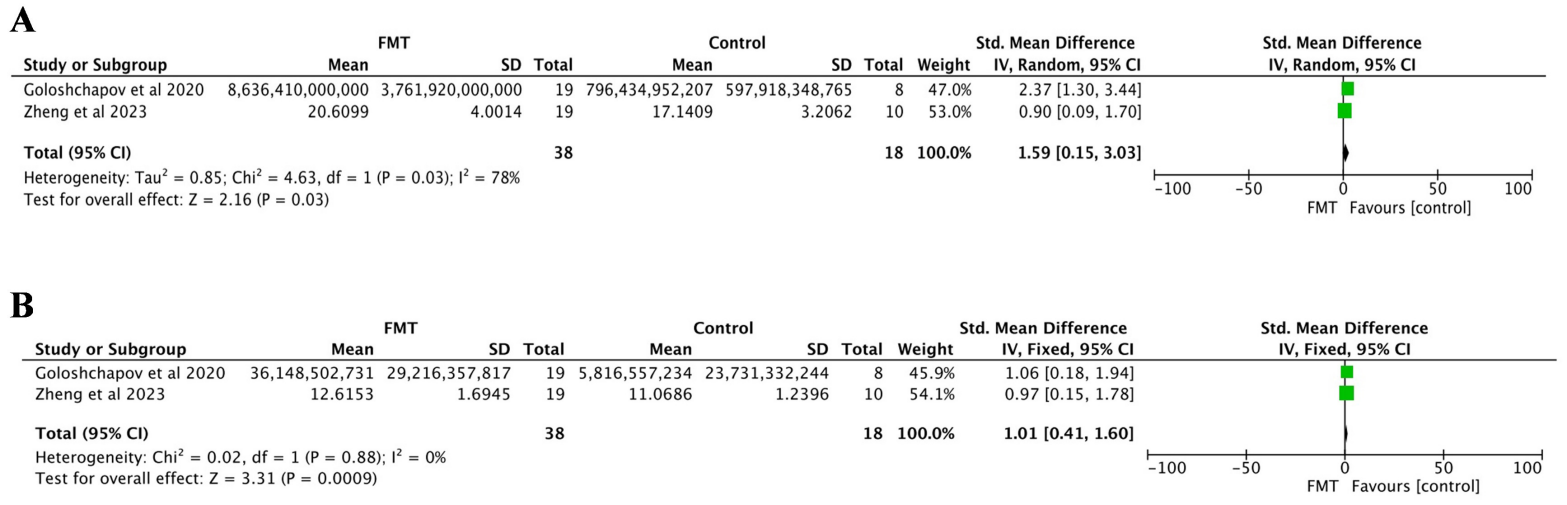
Gut microbiota changes analysis: **(A)** abundance of Bacteroides; **(B)** abundance of bifidobacterium.

Two studies were included, both reporting the abundance of Bacteroides (Zheng et al., 2023; Goloshchapov et al., 2020). The FMT group included 38 patients, and the control group consisted of 18 patients. Significant heterogeneity was noted (*P* = 0.03, *I*^2^ = 78%), and thus a random-effects model was applied. The meta-analysis indicated that the abundance of Bacteroides in the FMT group was significantly higher than that in the control group (SMD = 1.59, 95% CI = 0.15–3.03, *p* = 0.03; Figure 4A).

##### 3.3.2.7 Abundance of bifidobacterium

Two studies were included, both reporting the abundance of Bifidobacterium (Zheng et al., 2023; Goloshchapov et al., 2020). The FMT group comprised 38 patients, and the control group included 18 patients. No significant heterogeneity was detected (*p* = 0.88, *I*^2^ = 0%), and a fixed-effects model was used. The meta-analysis demonstrated that the abundance of Bifidobacterium in the FMT group was significantly higher than that in the control group (SMD = 1.01, 95% CI = 0.41–1.60, *p* = 0.0009; Figure 4B).

#### 3.3.3 Subgroup analyses

To explore potential sources of heterogeneity, subgroup analyses were performed for outcomes with significant heterogeneity.

##### 3.3.3.1 Incidence of aGVHD

**Figure 5:**
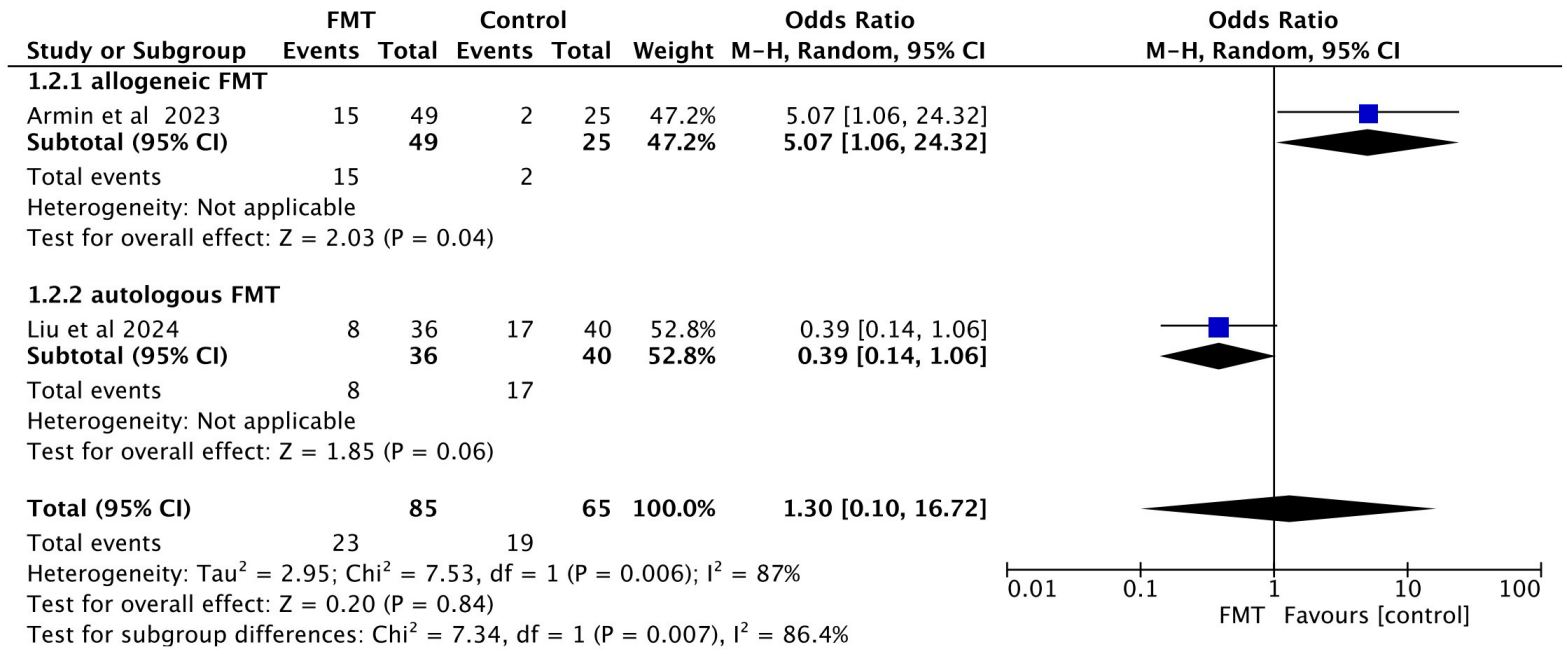
Subgroup analysis of aGVHD incidence by fecal microbiota source.

Stratification was performed according to the source of fecal microbiota (allogeneic FMT vs. autologous FMT). The results showed that in the allogeneic FMT group, the risk of aGVHD was higher in the FMT group than in the conventional regimen group (OR = 5.07, 95% CI = 1.06–24.32, *P* = 0.04); in the autologous FMT group, no statistically significant difference was observed between the two groups (OR = 0.39, 95% CI = 0.14–1.06, *P* = 0.06; Figure 5).

##### 3.3.3.2 Abundance of Bacteroides

**Figure 6:**
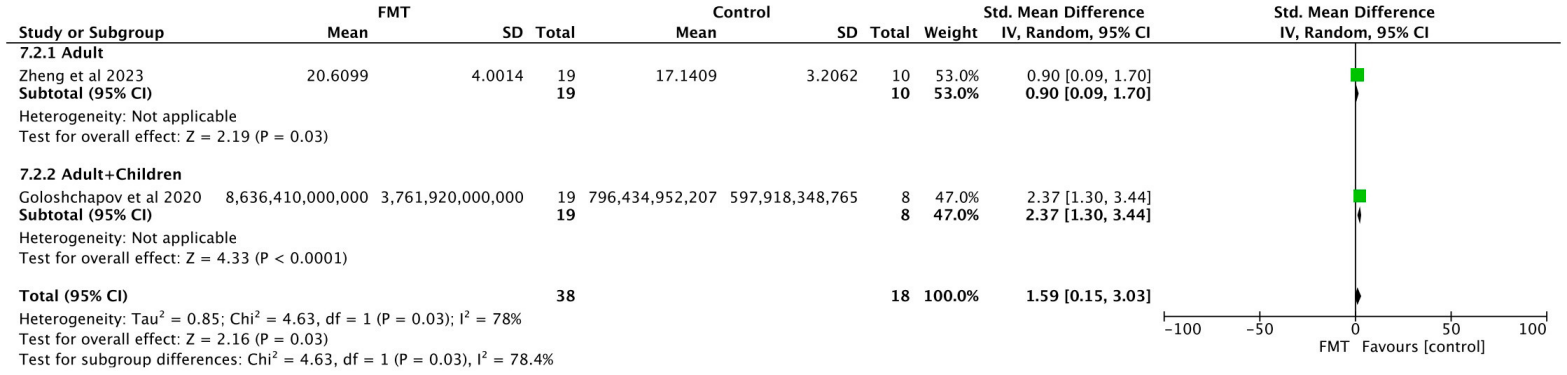
Subgroup analysis of bacteroides abundance by study population type.

Stratification was performed according to study population type (adult group vs. mixed pediatric and adult group). The results showed that the abundance of Bacteroides was significantly higher in the FMT group than in the conventional regimen group in both subgroups, which was consistent with the direction of the overall pooled effect (SMD = 0.90, 95% CI = 0.09–1.70, *P* = 0.03; SMD = 2.37, 95% CI = 1.30–3.44, *P* < 0.0001; Figure 6).

### 3.4 Sensitivity analysis

A sensitivity analysis was conducted by sequentially excluding each included study. The results indicated that no single study significantly influenced the overall outcomes, confirming the robustness of the meta-analysis findings.

### 3.5 Publication bias

**Figure 7:**
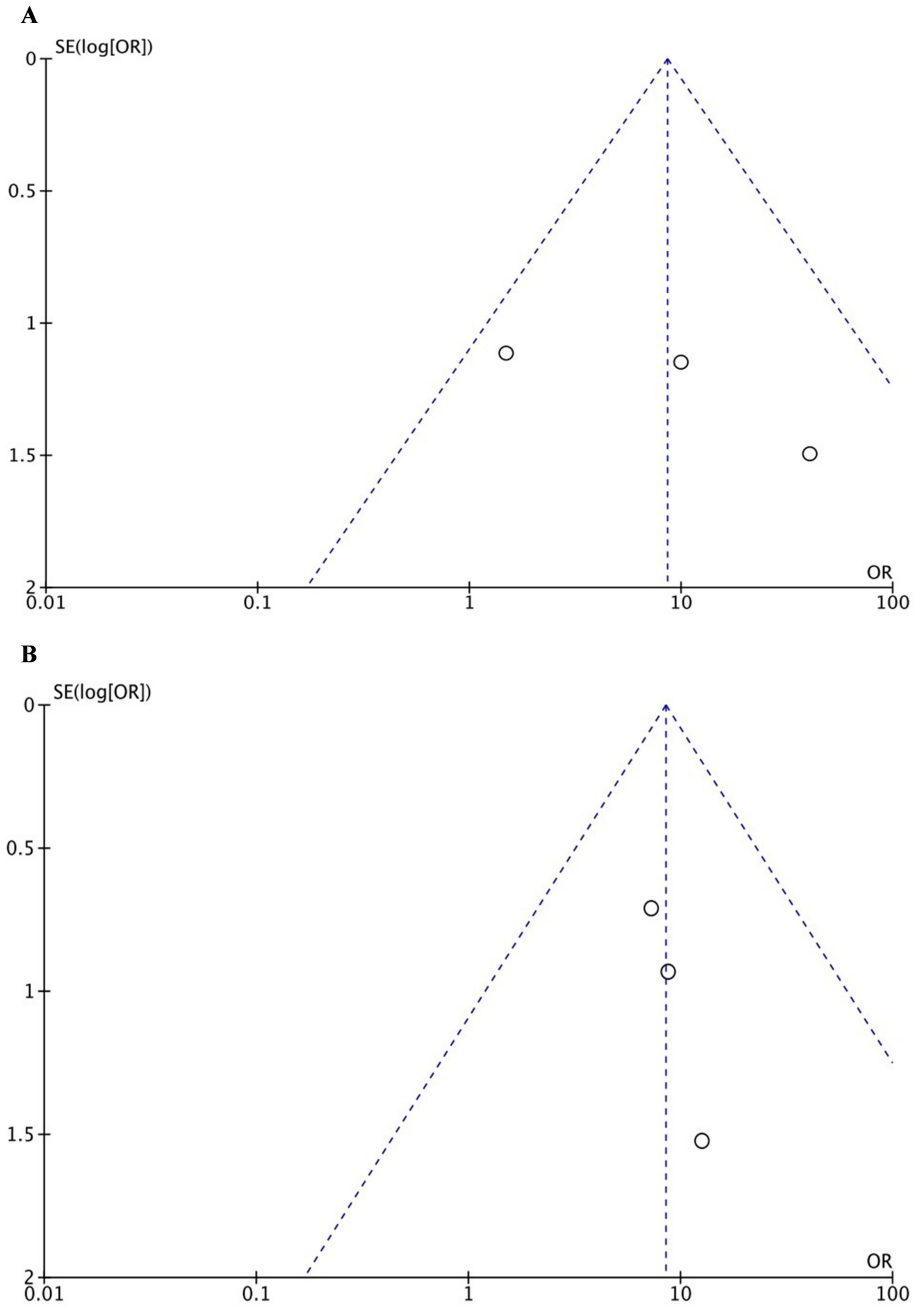
Funnel plots for publication bias: **(A)** 14-day complete response; **(B)** 30-day complete response.

For the CR at 14 and 30 days, the funnel plots exhibited approximate symmetry, with all included studies falling within the inverted funnel. Egger's test yielded P > 0.05, suggesting no detectable publication bias. However, due to the limited number of studies, the statistical power was insufficient, and a potential risk of publication bias cannot be ruled out (Figure 7).

### 3.6 Quality of evidence

The quality of evidence for mostly outcome measures was rated as moderate to high, supporting the reliability of the study findings. Nevertheless, the small number of included studies and their generally limited sample sizes affected the precision of the results. Therefore, future high-quality studies with larger sample sizes are warranted to validate these findings (Table 4).

**Table 4:** Certainty of evidence.

Outcome	Risk of bias	Inconsistency	Indirectness	Imprecision	Publication bias	Certainty of evidence
The incidence of aGVHD	Serious	Not serious	Not serious	Serious	None	Low
14-day CR of GI-aGVHD	Not serious	Not serious	Not serious	Not serious	Strong association	Moderate
30-day CR of GI-aGVHD	Not serious	Not serious	Not serious	Not serious	None	High
14-day clinical Response of GI-aGVHD	Not serious	Not serious	Not serious	Not serious	Strong association	Moderate
Bactermia	Serious	Not serious	Not serious	Not serious	None	Moderate
Sepsis	Serious	Not serious	Not serious	Not serious	None	Moderate
Bacteroides abundance	Not serious	Not serious	Not serious	Not serious	Strong association	Moderate
Bifidobacterium abundance	Not serious	Not serious	Not serious	Not serious	Strong association	Moderate

## 4 Discussion

aGVHD is an acute immune-mediated injury that occurs within 100 days after allo-HSCT. It results from the activation of donor-derived immune cells against host tissues, primarily targeting the skin, gastrointestinal tract, and liver (Jacobsohn and Vogelsang, 2007). Clinical studies indicate that aGVHD occurs in approximately 35-50% of transplant recipients, with gastrointestinal involvement being the most common manifestation. The mortality rate can reach as high as 55%, making aGVHD a leading cause of non-relapse mortality (Jacobsohn and Vogelsang, 2007; Borges et al., 2020). Therefore, improving the prevention and treatment of aGVHD is of significant clinical value for enhancing transplant success and patient prognosis.

The development and progression of aGVHD are closely associated with the pace and quality of immune reconstitution. The gut microbiota, often regarded as the body's “second immune system,” plays a crucial role in this process (Zhu et al., 2010; Shono and van den Brink, 2018). Certain commensals such as Lactobacillus, Bifidobacterium, and Bacteroides have been shown to modulate host immunity via the production of short-chain fatty acids, secondary bile acids, and indole derivatives, thereby limiting inflammatory responses and reducing aGVHD risk (Gao et al., 2018; Fuchs and Trauner, 2022; Fusco et al., 2023). However, pre-transplant conditioning regimens and broad-spectrum antibiotic use can compromise the intestinal mucosal barrier, disrupt the diversity and stability of the gut microbiota, and induce dysbiosis (Zama et al., 2017). This dysbiosis is often characterized by a reduction in beneficial bacteria such as Bifidobacterium and Bacteroides, alongside enrichment of potential pathogens like Enterococcus and Clostridioides difficile (Hayase et al., 2022). These factors lead to bacterial translocation, allowing pathogen-associated molecular patterns (PAMPs) and damage-associated molecular patterns (DAMPs) to be recognized by antigen-presenting cells (APCs), which in turn activate APCs and initiate donor T cell activation, proliferation, and a pro-inflammatory cytokine storm, thereby exacerbating tissue damage and promoting aGVHD progression (Lin et al., 2021). In addition, an imbalance in the gut microbiota has been shown to activate the inflammatory response by enhancing the expression of antigen-presenting molecules in small intestinal epithelial cells, among other mechanisms, thereby inducing aGVHD (Heuberger et al., 2024; Nguyen et al., 2025). Therefore, bacterial translocation and PAMPs are considered key mechanisms linking gut dysbiosis to the development of aGVHD (Lin et al., 2021; Han et al., 2020). FMT involves the transfer of healthy donor microbiota into the gastrointestinal tract of recipients with dysbiosis, aiming to rapidly restore microbial balance, repair the intestinal barrier, and reestablish immune homeostasis (Vindigni and Surawicz, 2017). Through this approach, FMT reduces bacterial translocation and PAMPs release, blocks downstream inflammatory cascades, and ultimately alleviates aGVHD-associated inflammation. Although FMT has demonstrated efficacy in patients with refractory GVHD (Youngster et al., 2024), its safety and efficacy for the prevention and treatment of aGVHD are still not well-established, prompting us to perform this meta-analysis.

Numerous studies support the critical role of gut microbiota in the prevention and treatment of aGVHD. Studies by Han et al. and Masetti et al. have indicated that enhancing gut microbial diversity and the abundance of specific beneficial taxa such as Bifidobacterium and Bacteroides can significantly reduce aGVHD risk (Han et al., 2020; Masetti et al., 2023; Sofi et al., 2021). Furthermore, research by Zheng et al. and Goloshchapov et al. also indicates that FMT has significant advantages in restoring the total amount and diversity of gut microbiota in patients with GI-aGVHD (Zheng et al., 2023; Goloshchapov et al., 2020). This study found that the abundances of Bifidobacterium and Bacteroides in the FMT group were significantly higher than in the conventional treatment group, corroborating the advantage of FMT in restoring microbial balance. Moreover, our study found that among patients with GI-aGVHD, the complete response rate and overall clinical effectiveness rate in the FMT group were significantly superior to those in the conventional regimen group, a finding consistent with the report by Han et al. (2020). This suggests that, for patients with GI-aGVHD who may respond poorly to conventional regimen, FMT could serve as an effective adjunctive or salvage treatment option. Regarding the prevention of aGVHD, existing research findings are inconsistent. This study found no significant difference in the incidence of aGVHD prevention between the FMT group and the conventional regimen group. However, research by Armin et al. suggests that after balancing factors such as the intensity of the preconditioning regimen, the incidence of aGVHD prevention in the FMT group was superior to that in the conventional regimen group (Rashidi et al., 2023). Therefore, we consider that maintaining gut microbiota balance may be a crucial aspect of aGVHD prevention and treatment. However, due to the limited number of studies currently included, the results require further validation through more prospective research.

Bloodstream infections represent another major factor affecting outcomes in allo-HSCT recipients (Taur et al., 2012). Whether FMT increases the risk of such infections remains a topic of interest. Studies by Montassier et al. suggest that maintaining gut microbiota diversity is a favorable factor for reducing the risk of bloodstream infections and that preoperative assessment of gut microbiota levels in allo-HSCT patients can help identify high-risk populations for such infections (Montassier et al., 2016). This study showed no significant difference in the incidence of bacteremia and sepsis between the FMT group and the conventional regimen group, indicating good safety profile of FMT. However, it is important to recognize that immunocompromised populations, such as allo-HSCT recipients, may be particularly vulnerable to the potential risks associated with FMT. Theoretically, there is a risk of transmitting multidrug-resistant organisms or opportunistic pathogens. Previous studies have reported adverse events following FMT, including Klebsiella pneumoniae infection, Clostridioides difficile infection, and Escherichia coli bacteremia (DeFilipp et al., 2019; Bier et al., 2023). Of greater concern, some studies have reported the transmission of multidrug-resistant Escherichia coli via FMT, resulting in fatal outcomes in immunocompromised recipients (DeFilipp et al., 2019), which may be related to factors such as donor screening, intensity of the preconditioning regimen, and administration methods (Bier et al., 2023; Rampanelli and Nieuwdorp, 2023; Airola et al., 2023). Although no such events were observed in the present study, these findings suggest that stringent donor screening, product evaluation, and standardized operational procedures are key to ensuring safety when applying FMT in immunocompromised populations, and should be carried forward into future research.

In recent years, in addition to FMT, a variety of microbiome-targeted therapeutic strategies have emerged. Among them, prebiotics, probiotics, and synbiotics have shown potential benefits in reducing the incidence of aGVHD by modulating the gut microbiota and immune responses (Yoshifuji et al., 2020; Rousseaux et al., 2023; Latif et al., 2023). Microbial consortia therapies (e.g., SER-155) are administered orally as specifically selected bacterial strains, aiming to inhibit pathogen colonization and reduce the risk of GVHD, with early clinical data demonstrating favorable safety and engraftment efficiency (Ponce et al., 2025). Microbiota-derived metabolites (e.g., short-chain fatty acids and desaminotyrosine) can directly regulate intestinal immunity and barrier function, among which desaminotyrosine (DAT) has shown the potential to protect intestinal stem cells and alleviate GVHD in preclinical models (Göttert et al., 2025). Precision probiotics emphasize selection based on strain-level functional differences (e.g., short-chain fatty acid production capacity), and studies have shown that optimally selected strain combinations outperform conventional probiotics in GVHD models (Ebigbo et al., 2025). In addition, preclinical studies have confirmed that bacteriophage-derived antibacterial enzymes can specifically lyse and eliminate intestinal Enterococcus faecium, thereby inhibiting the onset and progression of aGVHD (Fujimoto et al., 2024). Compared with these emerging therapies, FMT, as a complete microbial ecosystem, preserves the structural diversity and functional complexity of the donor microbiota and may exert more comprehensive and sustained modulatory effects on the recipient microbiota. In contrast, the emerging therapies offer theoretical advantages such as defined composition, high standardization, and low risk of pathogen transmission, demonstrating promising potential for clinical translation. Currently, direct comparative studies between FMT and these emerging therapies remain limited, and the relative efficacy and safety of these strategies in immunocompromised populations are still unclear. Future head-to-head studies are warranted to determine the optimal application scenarios for different microbiome-modulating interventions in allo-HSCT recipients.

This is the first meta-analysis systematically evaluating the safety and efficacy of FMT vs. conventional therapy for aGVHD prevention and treatment. It provides higher-level evidence supporting the use of FMT and highlights directions for future research. Nevertheless, several limitations should be acknowledged: (1) Geographic and ethnic bias: Among the six included studies, four were conducted in China, with limited data from other regions and populations. (2) Disease bias: Most studies focused on acute myeloid leukemia and acute lymphoblastic leukemia, with insufficient representation of other underlying diseases. (3) Limited sample size and missing patient characteristics: Only 262 patients were included, and details such as conditioning regimens and graft sources were not fully reported, precluding subgroup analyses to explore sources of heterogeneity. (4) Lack of long-term outcomes and dynamic microbiota data: The absence of longitudinal follow-up and functional microbiota analyses limits the evaluation of long-term FMT effects. Therefore, future multicenter, large-sample randomized controlled trials are warranted to validate and extend these findings.

## 5 Conclusion

FMT showed favorable effects in improving clinical symptoms of GI-aGVHD and increasing the abundance of beneficial gut bacteria, and no increased risk of bloodstream infection was observed. These findings suggest that, for patients with established GI-aGVHD who may respond poorly to conventional regimens (corticosteroids and/or immunosuppressants), FMT can serve as an effective adjunctive or salvage treatment. However, no significant advantage was observed for FMT in preventing aGVHD. Given the limited number of included studies, these findings should be interpreted as preliminary, and further high-quality research is warranted to confirm the efficacy and safety of FMT in this setting.

## Data Availability

The original contributions presented in the study are included in the article/supplementary material, further inquiries can be directed to the corresponding authors.
